# Knowledge, Attitudes, and Practices of Domestic Violence and Abuse (DVA) Among Healthcare Providers of a Tertiary Referral Hospital: A Cross-Sectional Study

**DOI:** 10.7759/cureus.98757

**Published:** 2025-12-08

**Authors:** Maimoona Ahmed, Vipin Anthony Das

**Affiliations:** 1 Obstetrics and Gynaecology, Fernandez Hospital, Hyderabad, IND; 2 Ophthalmology, Fernandez Hospital, Hyderabad, IND

**Keywords:** advocacy for women, attitude, domestic violence and abuse, healthcare staff, knowledge, practice, public health, survey

## Abstract

Introduction: Domestic violence and abuse (DVA) is often viewed as a social issue rather than a public health concern, despite evidence to the contrary. Survivors are more likely to seek help in hospitals than at police stations, making hospital staff crucial in identifying DVA.

Aim and objective: This study aimed to assess the knowledge, attitudes, and practices related to DVA among employees of a tertiary care hospital. A secondary objective was to determine the prevalence of DVA within this group.

Materials and methods:* *A prospective, survey-based study was conducted among all employees of a tertiary maternity center. An online questionnaire assessing DVA-related knowledge, attitudes, and practices was distributed, with anonymous responses collected. Additional questions explored the prevalence of DVA among respondents. Results were reported as percentages.

Results: Of 1,683 employees, 1,005 responded (60%). Most respondents were women aged 18-30 years (84.78%). Healthcare providers comprised 57.61%, followed by administrative (11.64%), paramedical (10.45%), and support staff (20.3%). Responses revealed persistent misconceptions and patriarchal influences surrounding DVA. Overall, 25.93% reported experiencing DVA, and 46.45% had witnessed DVA in their close environment.

Conclusion:* *The study highlights existing gaps in knowledge and attitudes toward DVA within a maternity care setting and shows that healthcare providers themselves are not immune to interpersonal violence. Regular training, sensitization, and routine DVA screening during hospital visits are essential to ensure timely care, accurate documentation, and appropriate referrals for survivors.

## Introduction

Domestic violence and abuse (DVA) is one of the most pervasive forms of gender-based violence against women. It is defined as a pattern of controlling, coercive, threatening, degrading, and violent behavior, including sexual violence by a partner/ex-partner, in-laws, parents, siblings, and other relatives [1]. Globally, one in six women is a victim of DVA [2]. In India, the National Family Health Survey, NFHS-4, estimates one in three married women to be subjected to DVA, with physical violence being the most common (30%), followed by emotional violence (14%) and sexual violence (7%) [3]. These numbers are the tip of the iceberg, as 9 out of 10 women do not report the abuse [3]. Several factors perpetuate DVA, such as traditional beliefs of male superiority, cultural acceptance of domestic violence as a private affair, and societal normalization of violence to resolve discords [4].

There is growing evidence that DVA leads to adverse effects on women’s physical, mental, and reproductive health [5]. Physical problems include injuries, sexually transmitted diseases, urinary tract infections, and pelvic inflammatory diseases. Psychological issues such as depression, anxiety, low self-esteem, psychosomatic complaints, substance abuse, suicidal thoughts, and thoughts of self-harm are also attributed to violence and abuse against women [5]. DVA is also associated with poor pregnancy outcomes such as anemia, stillbirth, placental abruption, fetal injury, preterm delivery, and low birth weight [6]. DVA incidence tends to increase during pregnancy, with the pregnancy itself being a typical trigger for the abuser. Low socioeconomic status, partner alcohol misuse, stress, and partner jealousy are all risk factors for violence during pregnancy [7].

The medical community plays a vital role in identifying women who experience DVA and halting the cycle of abuse through screening, offering support, and referring to other specialist services. Abused women are much more likely to be in contact with healthcare services than the police [8]. However, the role of healthcare providers and health systems in responding to sexual assault has been plagued by biases and insensitivity. In many cases, the healthcare providers do not recognize the signs of DVA due to a lack of training and understanding of the problem [9].

With this background, we conducted a survey-based study on the knowledge, attitudes, and practices regarding DVA at our tertiary care referral center. As we cater to women and children, we needed to know the gaps in our own understanding of this public health problem. As a secondary objective, we also noted the prevalence of this social evil among our own employees.

## Materials and methods

Study design

This was a prospective cross-sectional survey-based study conducted over 2 months from August to September 2024. It was administered to the employees of a tertiary care referral hospital catering to women’s health, maternity, and newborn care. The participants included all employees of the hospital, including clinical and non-clinical staff. The clinical staff included doctors, midwives, and nursing teams, along with paramedical services. The non-clinical staff included administrative services, housekeeping, security, and other departments.

The survey was divided into three sections. The first section included demographic details of the participants. The second was the questionnaire on knowledge, attitudes, and practices pertaining to DVA. There were eight questions on knowledge, six on attitudes, and five questions on practice parameters when dealing with DVA. This structured questionnaire was developed from a literature review and observations. The third section aimed to understand the prevalence of DVA among employees and those in their close social circles, such as friends and family.

Data collection and analysis

The survey was distributed online to all the employees on an internal channel via email and WhatsApp. The participants gave implied consent by completing the online questionnaire. The responses were collected anonymously. All the data obtained were entered into Microsoft Excel (Microsoft Corp., Redmond, WA, USA) spreadsheets, and the results were expressed as percentages. Continuous variables were summarized using measures such as means and standard deviations, while categorical variables were summarized using frequencies and percentages. The data were represented using tables and appropriate diagrams such as bar diagrams, pie diagrams, and box plots.

Ethical committee clearance was obtained from the FHERF IRB (EC reference no.64_2025). 

## Results

The study covered a total of 1,683 employees at the hospital at the time of the survey, and 1,005 responded, leading to a response rate of 59.7%.

Demographics

The demographic profile of the survey participants has been detailed in Table 1. A majority of the respondents were female, reflecting the center’s large female workforce. Young nurses in the age group of 18-30 years formed a big part of the group.

**Table 1:** Demographic profile The data has been represented as N (%).

Demographics			Number (N)	Percentage (%)
Gender		Male	150	15
		Female	855	85
Age		18-30 years	564	56.02
		31-40 years	275	27.36
		41-50 years	129	12.84
		51-60 years	34	3.48
		>60 years	3	0.3
Occupation		Doctor	127	12.64
		Midwife	36	3.58
		Nurse	416	41.39
		Paramedical	105	10.45
		Administrative	117	11.64
		Others (housekeeping, security, and patient assistance)	204	20.3

Knowledge

There was a total of eight questions pertaining to knowledge regarding DVA.

Warning signs that a woman may have been subjected to sexual violence or domestic violence: Most responded as yes to the clinical signs and symptoms such as depression/anxiety/stress (70.8%), thoughts and plans of self-harm/suicide (70.7%), frequent injuries (60.3%), repeated hospital visits (59%), repeated sexually transmitted infections (58%), and chronic unexplained pain (58%). Fewer were aware that other symptoms such as repeated miscarriages/termination of pregnancy (56%), repeated urinary tract infections (49%), preterm birth (39.5%), and stillbirth (36.9%) are also some of the presentations of DVA.

Table 2 depicts the responses regarding which of the following comes under the Domestic Violence and Abuse Act, 2005.

**Table 2:** Types of abuse covered in the Domestic Violence and Abuse Act, 2005 The data are represented as N (%).

Which of the following comes under the Domestic Violence and Abuse Act, 2025	Yes	No
Physical abuse	868 (86.4%)	137 (13.6%)
Sexual abuse	844 (83.9%)	161 (16.1%)
Verbal abuse	803 (79.9%)	202 (20.1%)
Emotional abuse	810 (80.6%)	195 (19.4%)
Economic abuse	690 (68.6%)	315 (31.4%)

**Figure 1:**
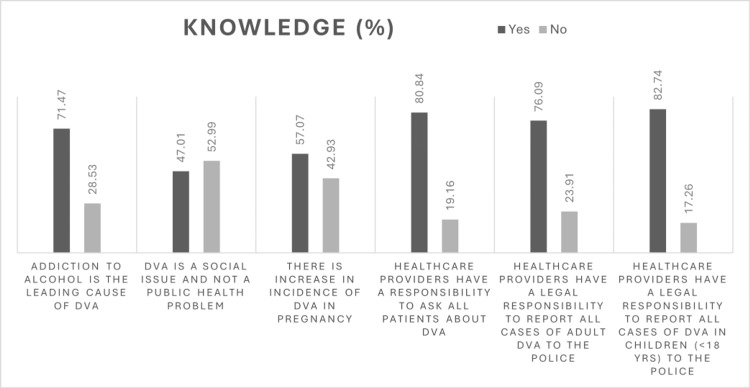
Knowledge regarding DVA The data are represented as %. DVA: Domestic violence and abuse.

Figure 1 depicts the responses to the remaining six questions covering the knowledge domain.

Attitude

The second section covered items that assessed personal attitude toward DVA. There was a total of six questions in this domain.

**Figure 2:**
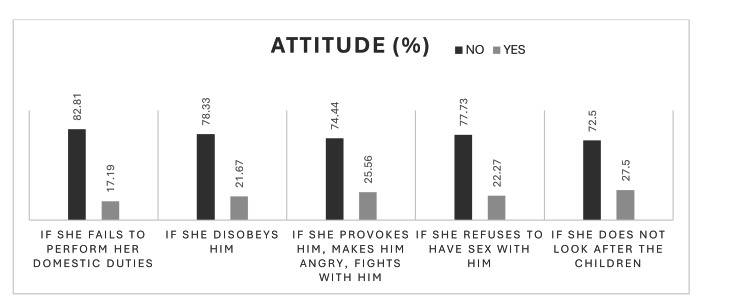
Attitude regarding DVA: “Do you think it is acceptable for a man to abuse his wife or partner in the following situations?” The data are represented as %. DVA: Domestic violence and abuse.

Figure 2 depicts responses to the question “Do you think it is acceptable for a man to abuse his wife or partner in the following situations?”

A sub-analysis of the "yes" responses with comparison of the demographic profile was performed. Over 80% of respondents were female, and 60%-65% were in the age group of 18-30 years. About 50% of respondents were from the nursing team, 25% from the other groups, followed by up to 15% from the paramedical team. Around 4% of doctors also responded yes to the above statements.

**Figure 3:**
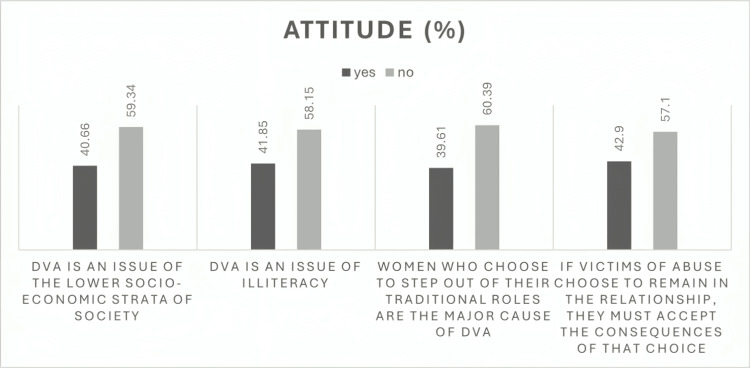
Attitude regarding DVA The data are represented as %. DVA: Domestic violence and abuse.

Figure 3 depicts responses to the other four questions in the attitude section.

We performed a sub-analysis of the “yes” responses and compared the associated demographic characteristics. Over 85% of respondents were female, and 51%-62% were in the age group of 18-30 years. About 47% of respondents were from the nursing team, 18% from the paramedical team, and 10% from the administrative staff. Around 5% of doctors also responded yes to the above statements.

The last question related to attitude covered asking questions about DVA. Table 3 details the responses received.

**Table 3:** Attitude toward asking questions about DVA DVA: Domestic violence and abuse.

Statement	Yes (%)	No (%)
Asking patients about domestic violence is an invasion of their privacy	43.65	56.35
If I ask a non-abused patient about DVA, they will get angry	52.77	47.23
The way a couple chooses to resolve a conflict is not my business	46.79	53.21
I do not know how to help or what to do if a woman discloses a history of DVA	46.64	53.36

Practice

**Figure 4:**
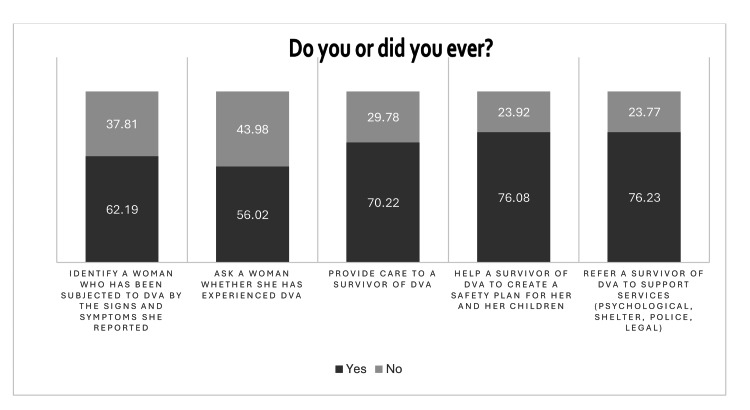
Practice related to DVA The data are represented as %. DVA: Domestic violence and abuse.

The responses to the five questions related to practice regarding DVA are provided in Figure 4.

Prevalence of DVA

We also asked direct questions to look for the prevalence of DVA among our cohort of employees.

**Figure 5:**
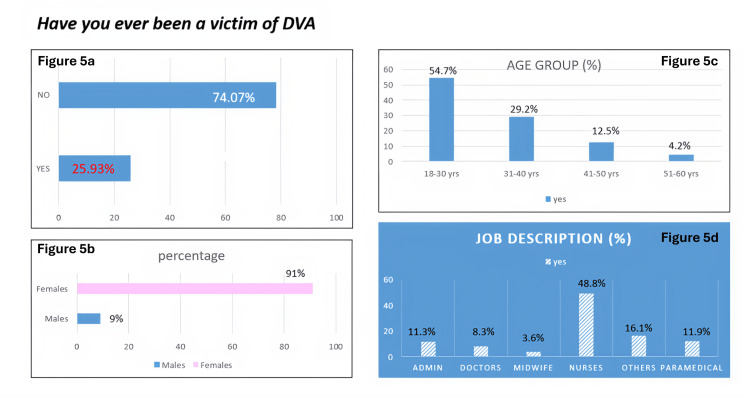
Prevalence of DVA among employees The data are presented as %. Figure 5a: Response to the question "Have you ever been a victim of DVA?" Figure 5b: Gender distribution of "yes" responses Figure 5c: Age distribution of "yes" responses Figure 5d: Occupation distribution of "yes" responses DVA: Domestic violence and abuse.

Figure 5 depicts the responses to the question "Have you ever been a victim of DVA?" It also shows the demographic distribution of the employees that responded "yes" to the question.

**Figure 6:**
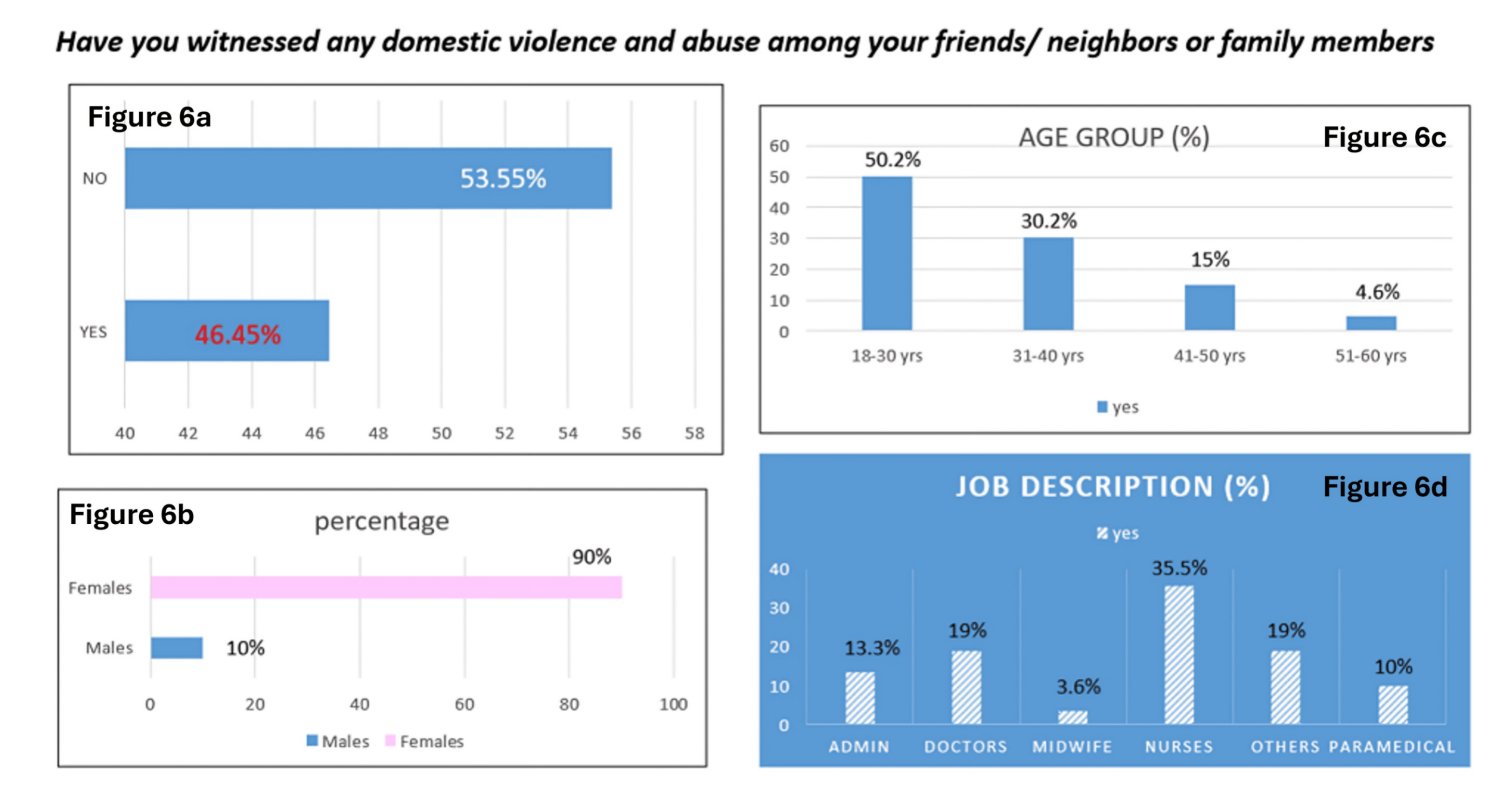
Prevalence of DVA among social circle of employees The data are presented as %. Figure 6a: Response to the question "Have you ever witnessed DVA among friends, neighbors, or family members?" Figure 6b: Gender distribution of "yes" responses Figure 6c: Age distribution of "yes" responses Figure 6d: Occupation distribution of "yes" responses DVA: Domestic violence and abuse.

Figure 6 shows the responses to the question "Have you witnessed any DVA among your friends, neighbors, or family members?" Further sub-analysis of the positive response is also represented in the figure.

## Discussion

Our study assessed the knowledge, attitudes, and practices regarding DVA among employees in a tertiary care referral hospital catering to women and children. With a response rate of nearly 60%, the results provide valuable insights into awareness and preparedness among healthcare providers and support staff in identifying and responding to DVA.

Knowledge about DVA

Overall, participants demonstrated moderate knowledge regarding the warning signs and legal aspects of DVA. The majority correctly identified clinical indicators such as depression, self-harm, and recurrent injuries, aligning with a study by Arora et al. reporting similar awareness patterns among healthcare providers [10]. However, the knowledge of less obvious clinical manifestations, such as recurrent miscarriage, urinary tract infections, or stillbirth, was limited, echoing findings from other studies, where awareness of reproductive and obstetric indicators of abuse was also poor [11,12]. While most respondents recognized physical, sexual, verbal, and emotional abuse as forms of DVA, awareness of economic abuse was lower (68.6%). Similar trends have been observed in other studies, where financial control or deprivation was often under-recognized as abuse [12,13]. This gap suggests the need for focused training on the multidimensional nature of DVA as defined under the Protection of Women from Domestic Violence Act (PWDVA), 2005 [14].

Attitude toward domestic violence

Attitudinal findings revealed persistent misconceptions and normalization of abuse among some respondents. Despite most being young and female, 40%-50% believed asking about DVA could invade privacy or provoke patient anger. These results are concerning but consistent with research from other hospitals showing reluctance to inquire about abuse due to fear of offending patients, time constraints, and lack of confidence [10,15]. Interestingly, a small but significant proportion of participants (especially nurses and paramedical staff aged 18-30 years) considered wife beating acceptable in certain situations. Such attitudes reflect entrenched sociocultural beliefs about gender roles and marital privacy, which have been documented as barriers to effective DVA intervention in healthcare settings [15,16]. Targeted sensitization programs emphasizing gender equality and human rights are therefore essential.

Practice regarding DVA

In the practice domain, while data show some awareness of screening and reporting mechanisms, actual implementation remains suboptimal. The majority of the respondents were hesitant to initiate conversations regarding DVA. This aligns with evidence that even when knowledge exists, translation into practice is often hindered by a lack of institutional support, unclear protocols, and fear of legal repercussions [9]. Studies from an Indian tertiary hospital have shown that less than one-third of healthcare providers routinely ask patients about DVA, largely due to inadequate training and referral systems [10]. Other barriers cited are concern about offending the patient, embarrassment about bringing up the topic, and the patient being accompanied by a partner or children [12]. Encouragingly, however, a recent survey found that providers who received domestic violence education were more likely to have screened their patients and more likely to take action when DVA was suspected [17]. This further underscores the need for training and sensitization programs for healthcare providers and other frontline healthcare personnel.

Prevalence of DVA

Our study also explored the prevalence of DVA among the employees and found that 25.9% of respondents reported having personally experienced DVA. Additionally, nearly half (46.5%) had witnessed DVA among friends, neighbors, or family members. These findings indicate that DVA is a significant issue even among healthcare providers and individuals who are often viewed as being more aware of health and social issues.

The prevalence observed (25.9%) in this study aligns with prior research indicating that healthcare providers are not immune to interpersonal violence. Global estimates of lifetime intimate partner violence among women range from 27% to 33% [18]. A study conducted by Dheensa et al. among healthcare providers in various settings has reported prevalence rates between 20% and 45% [19]. The similarity of our findings to these figures suggests that DVA among healthcare providers mirrors the patterns observed in the general population. The predominance of female respondents among younger age groups (18-30 years) who reported victimization may reflect both the demographic composition of the hospital workforce and the heightened vulnerability of younger women to domestic violence [20]. The nursing team had the highest proportion of DVA victims (48.8%), consistent with other studies identifying nurses as a high-risk group due to gender distribution, stress, and shiftwork patterns [21].

Almost 46% of participants reported having witnessed DVA in their social circles, which underscores the pervasiveness of the problem and the normalization of violence within communities. The implications of these findings are significant. Healthcare providers who experience or witness DVA may face emotional and occupational consequences, including burnout, absenteeism, and decreased work performance [19]. Moreover, unaddressed personal experiences with DVA may affect healthcare providers’ ability to identify and support patients experiencing similar abuse [22]. These findings highlight the need for institutional policies that provide confidential support, counseling services, and awareness training within healthcare organizations.

Implications for policy and training

Given that our institution caters primarily to women and children, a population highly vulnerable to abuse, the findings highlight the need for structured training modules, clear reporting pathways, and multidisciplinary response teams. Routine screening for DVA during antenatal, gynecological, and pediatric consultations, combined with confidential counseling, could significantly improve detection and support [17]. Obstetricians and gynecologists are in a unique position to assist survivors of DVA because of the nature of their relationship with their patients. They have multiple opportunities for interventions during their annual examinations, family planning, pregnancy, and follow-up visits for ongoing care. Screening women at different times is important, as women do not disclose the abuse during the initial few checks [8]. Periodic training workshops can address attitudinal barriers and improve confidence among healthcare providers in handling disclosures.

The prevalence findings have important implications for healthcare organizations in India. PWDVA [14] provides a comprehensive legal framework for supporting survivors, yet awareness and implementation remain limited within workplaces. Hospitals such as ours, as major employers of women, are well-positioned to integrate DVA screening, counseling, and referral pathways into employee wellness programs. The National Health Policy also emphasizes gender equity and safe working environments for healthcare providers, suggesting an institutional obligation to address DVA as both a public health and occupational welfare concern [23]. Establishing confidential reporting mechanisms, psychological support services, and regular sensitization workshops can help build a safer and more supportive workplace culture.

Raahat Crisis Centre

To address the gaps identified in the study, our institute established a hospital-based crisis center, the first of its kind in a private hospital, for survivors of DVA. The center provides compassionate, confidential, and coordinated support, including clinical care, counseling, legal and police referrals, skill-building opportunities, and livelihood assistance. In addition to direct survivor services, the center prioritizes training and sensitization of healthcare providers and other hospital staff. It also conducts community awareness campaigns to promote understanding and prevention of DVA. Through these integrated efforts, the center aims not only to support individual survivors but also to position DVA as a critical public-health challenge, one that demands a proactive, system-wide response to promote safety, healing, and long-term societal change.

Strengths and limitations

The large and diverse sample of healthcare staff strengthens the reliability of the findings. However, the self-reported nature of responses and potential social desirability bias may have influenced results, particularly for attitude-related items. Additionally, findings from a single tertiary care hospital may not be generalizable to smaller facilities or rural healthcare settings.

This study also provides valuable insight into DVA prevalence among a diverse hospital workforce. Underreporting is likely, given the sensitivity and stigma surrounding DVA, suggesting that true prevalence may be even higher. Future research using qualitative methods could provide a deeper understanding of the contextual and occupational factors influencing DVA risk among healthcare providers.

## Conclusions

This study highlights the important gaps in knowledge, attitudes, and practices related to DVA among healthcare providers in a tertiary care referral center. While awareness of common signs and forms of violence is relatively high, misconceptions and limited practical engagement persist. Addressing these through regular sensitization, inclusion of DVA modules in medical and nursing curricula, and institutional support for screening and reporting will be crucial to improving the healthcare response to DVA in India. The prevalence of DVA among hospital employees in this study is concerningly high, with young female nurses being the most affected group. Given the potential personal and professional impacts, healthcare institutions should adopt proactive measures to promote awareness, provide safe reporting mechanisms, and ensure access to psychological and legal support for affected staff.

## Appendices

Knowledge, attitudes, and practices for DVA survey

Demographic Details

**Table 4:** Demographic details

Gender	
Male	
Female	
Age	
18-30 years	
31-40 years	
41-50 years	
51-60 years	
>60 years	
Area of work	
Doctor	
Midwife	
Nurse	
Paramedical staff	
Admin services	
Security	
Housekeeping	

**Table 5:** Knowledge regarding DVA

Knowledge		Yes	No
	Which of the following are warning signs that a woman may have been subjected to sexual violence or domestic violence?		
	Repeated miscarriages or termination of pregnancy		
	Repeated sexually transmitted infections		
	Repeated urinary tract infections		
	Chronic unexplained pain or conditions (e.g., pelvic, headaches)		
	Frequent injuries		
	Preterm birth		
	Stillbirth		
	Depression, anxiety, or chronic stress		
	Thoughts, plans or acts of self-harm, or (attempted) suicide		
	Repeated visits to the hospital with no clear diagnosis		
	Which of the following come under the Domestic Violence and Abuse Act		
	Physical abuse		
	Sexual abuse		
	Verbal abuse		
	Emotional abuse		
	Economic abuse		
	Addiction to alcohol is a leading cause of DVA.		
	DVA is a social issue and not a public health problem.		
	There is increase in incidence of DVA in pregnancy.		
	Health care providers have a responsibility to ask all patients about DVA.		
	Health care providers have a legal responsibility to report all cases of adult DVA to the police.		
	Health care providers have a legal responsibility to report all cases of DVA in children (<18 yrs) to the police.		

**Table 6:** Attitude regarding DVA

Attitude	Acceptability of domestic violence and abuse	YES	NO
	Do you think it is acceptable for a man to abuse his wife or partner in the following situations:		
	If she fails to perform her domestic duties		
	If she disobeys him		
	If she provokes him or makes him very angry or fights with him		
	If she refuses to have sex with him		
	If she does not look after the children		
	If he suspects that she is being unfaithful		
	DVA is an issue of lower socio-economic strata of society.		
	DVA is an issue of illiteracy.		
	Women who choose to step out of traditional roles are the major cause of DVA.		
	If victims of abuse choose to remain in the relationship after repeated episodes of abuse, they must accept consequences of that choice.		
	Attitude influencing decisions to ask about domestic violence and abuse		
	Asking women about domestic violence is an invasion of their privacy.		
	If I ask non-abused women about domestic violence, they will get very angry.		
	The way a couple chooses to resolve a conflict is not my business.		
	I do not know how to help or what advice to give if a woman discloses DVA.		

**Table 7:** Practice regarding DVA

Practice	Do you or did you ever?	YES	NO
	Identify a woman who is or has been subjected to domestic violence by signs and symptoms.		
	Ask a woman about whether she has experienced domestic violence.		
	Provide care to a woman who is or has been subjected to domestic violence.		
	Help the woman to create a plan to increase her and her children’s safety.		
	Refer the woman to support services available (psychological, legal, shelter, etc.)		

Prevalence of DVA

**Table 8:** Prevalence of DVA

	YES	NO
Have you ever been a victim of domestic violence and abuse?		
Have you witnessed any domestic violence and abuse among your friends/neighbors or family members?		
